# A 24-month randomized clinical study of flowable bulk-fill resin composites in Class III restorations

**DOI:** 10.4317/jced.61153

**Published:** 2024-02-01

**Authors:** Antonio Signore, Luca Solimei, Marianna-Georgievna Arakelyan, Stefano Benedicenti, Cristina Mollica

**Affiliations:** 1 Therapeutic Dentistry Department, Institute of Dentistry, I.M. Sechenov First Moscow State Medical University, Moscow, Russian Federation; 2 Department of Surgical and Diagnostic Sciences (D.I.S.C.), University of Genoa, Genoa, Italy; 3 Department of Statistical Sciences, Sapienza University of Rome

## Abstract

**Background:**

The 24-month clinical performance of 3 commercially available flowable bulk-fill resin composites in Class III restorations was evaluated.

**Material and Methods:**

Forty-two patients, 27 females (64.3%) and 15 (35.7%) males, received at least 3 Class III restorations that never exceeded 4 mm depth and width. One hundred thirty-eight teeth, divided up into three groups (n=46), were randomly restored with Admira Fusion x-base (AFB), Estelite Bulk Fill Flow (EBF) and SDR flow+ (SDR) in one single increment. A 2-step self-etch adhesive system Clearfil SE Bond (C-SE) with selective enamel etching was used for all restorations. The restorations were clinically evaluated using a slightly modified USPHS criteria at the baseline and every 6 months for 24 months. Success rates of each material between the baseline and at 24 months were compared with the McNemar test. At each timepoint, the comparison of the clinical performance among materials in terms of the ratings of the considered criteria was analyzed by using the Friedman and Cochran’s Q tests.

**Results:**

At the end of the 24-month follow-up, the overall clinical success rate was 100% for each tested material. However, significant differences among the composites were highlighted for several criteria involving the marginal adaptation, marginal discoloration, surface texture, surface staining and color match.

**Conclusions:**

The flowable bulk-fill resin composites tested showed an overall good effectiveness for Class III restorations after the 24 months, although significant rating differences among the materials emerged for some specific clinical criteria.

** Key words:**Bulk-fill, Class III restoration, flowable composite, clinical study, self-etching adhesive.

## Introduction

Recent development of effective bonding strategies and resin composites materials offer the major advantage of preserving dental hard tissues, complying with the consolidated concept of a conservative restorative approach (1,2). Currently various resin composites such as microhybrid resin composites and the so-called nanofilled composites, have been recommended for direct anterior restorations (3,4). Several clinical trials have reported satisfactory clinical performance, high survival rates and achievement of esthetic outcomes in anterior teeth (3). The effectiveness of such a treatment is in terms of esthetic, function and longevity has been confirmed in systematic reviews of clinical trials on anterior resin composite restorations (3,5,6). A high clinical success rate for Class III restorations was reported in previous short-term clinical investigations (7,8).

However, these conventional composite materials need to be placed in the cavity with an incremental technique, thus all increments must be light-cured separately. Incremental layering techniques were recommended in order to reduce the final volumetric polymerization shrinkage stress (post-gel shrinkage), therefore, to minimize marginal and internal interface gap formation and to avoid depth-of-cure limitations (9). Manufacturers of contemporary restorative composites offer a large number of chromatic shades with different opacities and translucencies for replacing dentin and enamel. In fact, the multilayer technique also allows the clinician to apply dentin-shaded resin composite and enamel-shaded resin composite to mimic the optical properties of natural teeth (9,10). Nevertheless, this incremental technique makes the restorative procedure more time consuming and the placement of layers in small preparations is considered technique-sensitive.

More recently, the continuous search for a low shrinkage resin composites has led to the introduction of “bulk-fill” resin composites. This new composite materials do not require the incremental technique and can be applied in bulk up to a thickness of 4mm, having an adequate polymerization, thus meeting the clinician’s desire for more simplified and faster restorative procedures in clinical daily practice (11). Low polymerization shrinkage was achieved changing monomer chemistry or structure, increasing the molecular weight per reactive group, increasing the filler load, modifying photoinitiation systems and incorporating monomers that act as modulators of the polymerization reaction (11,12). Moreover, due to a lower content of light absorbing pigments, mainly inorganic iron oxides, these materials are more translucent, allowing greater light dissipation through the material (13,14).

To date two different types of bulk-fill resin composites are commercially available: the full-body bulk-fill and the base, also referred to as flowable bulk-fill resin composites (15). The full-body bulk-fill composites have a high inorganic filler loading, resulting in a high viscous consistency. For this reason, they are also referred to as paste-like or sculptable bulk-fill resin composites. Their resulting volume analysis and shrinkage stress showed promising outcomes compared with regular methacrylate composites, the flexural strength and wear resistance have been compared to those of conventional resin composites and the creep has been reported as adequate (16-18). This may support the intended use of these materials for bulk filling in areas of high functional load.

On the other hand, flowable bulk-fill resin composites have mostly a lower inorganic filler content, than full-body bulk-fill (15). It has been speculated that due to a lower viscosity they can facilitate the adaptation of the material on the cavity walls and can be applied more easily in small cavities by means of a syringe tip. However, inferior mechanical properties have been reported for low-viscosity bulk-fill composites compared to highly filled nanohybrid composite, which may warrant caution for their application in highly loaded areas (19,20). In accordance with these findings, some recent studies showed a lower wear resistance and strength, thus suggesting the placement of a capping layer made of a high-viscosity conventional composite for restorations under high occlusal load (17). Flowable bulk-fill resin composites are commonly used as a base, followed by capping with the conventional resin composites, and recent clinical evaluations showed acceptable clinical effectiveness in Class I and II cavities (15,21). Furthermore, concerns have been raised in using this kind of restorative resin composites without a conventional composite at the restoration surface (15,18,22).

Most recently, a few manufacturers claim that flowable bulk-fill resin composites are suitable for being used in Class III restorations without an additional capping layer. The potential advantage of this new technique might be a more simplified and faster restorative procedure in clinical daily practice. Therefore, the objective of the present study was to evaluate the 24-month clinical performance of 3 commercially available flowable bulk-fill resin composites in Class III restorations without any additional capping layer.

## Material and Methods

Patients attending the Department of Department name of the University of university name (Country) requiring no less than 3 Class III cavity restorations were recruited for this study during the period from November 2020 to April 2021. The study protocol was conducted after prior review and approval by the local Ethical Committee (protocol number: 1727/2020) and performed in accordance with the Declaration of Helsinki, as well as following the guidelines of Consolidated Standards of Reporting Trials (CONSORT) (23).

First examination, restorative procedures and recall visits were performed according to National Health Regulation for Infection Prevention and Control during health care of coronavirus disease (‎COVID-19)‎. No monetary compensation was provided for the participation.

In this randomized, single center clinical study, 3 flowable bulk-fill restorative resin composites were compared: Admira Fusion x-base (Voco; Cuxhaven, Germany); Estelite Bulk Fill Flow (Tokuyama Dental Corporation, Tokyo, Japan) and SDR flow+ Bulk Fill Flowable (Dentsply Sirona, Milford, DE, USA). The materials used and their compositions are given in Table 1.

**Table 1:**
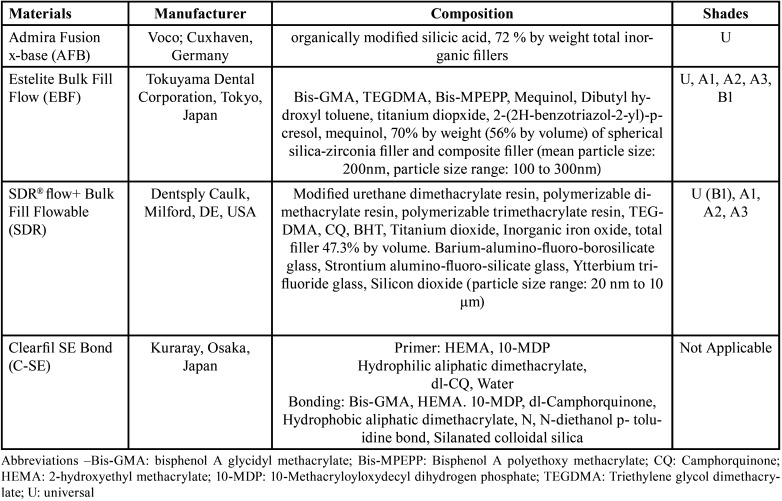
Materials, composition and shades according to the information supplied in the safety data sheets and manufacturer’s instruction.

The following null hypotheses were tested: 1) for each material, there is no difference in terms of overall clinical success rate (i.e. occurrence of Alpha or Bravo score) after 24 months from the baseline; 2) for each material, there is no difference in terms of the ratings of the clinical criteria after 24 months from the baseline and 3) at each time point, there is no difference among the materials in terms of the ratings of clinical criteria. Unlike the first null hypothesis, for the second and third null hypotheses the ratings Alpha and Bravo are not considered equivalent, but as distinct decreasing ordinal scores.

Concerning the sample size computation for evaluating the above hypotheses, we considered the F-test for a repeated measures ANOVA with 3 within-subject conditions. By using the G*Power software, we determined that a total of 42 measurements in each group is the minimum sample size to detect a difference of 0.25 in the median ratings (medium effect size) between pairs of materials with a power of 0.95 at the conventional 0.05 level of significance. Indeed, the number of restorations considered in our study is even larger (n=46) to take possible dropouts into account.

-Study population and randomization.

**Figure 1:**
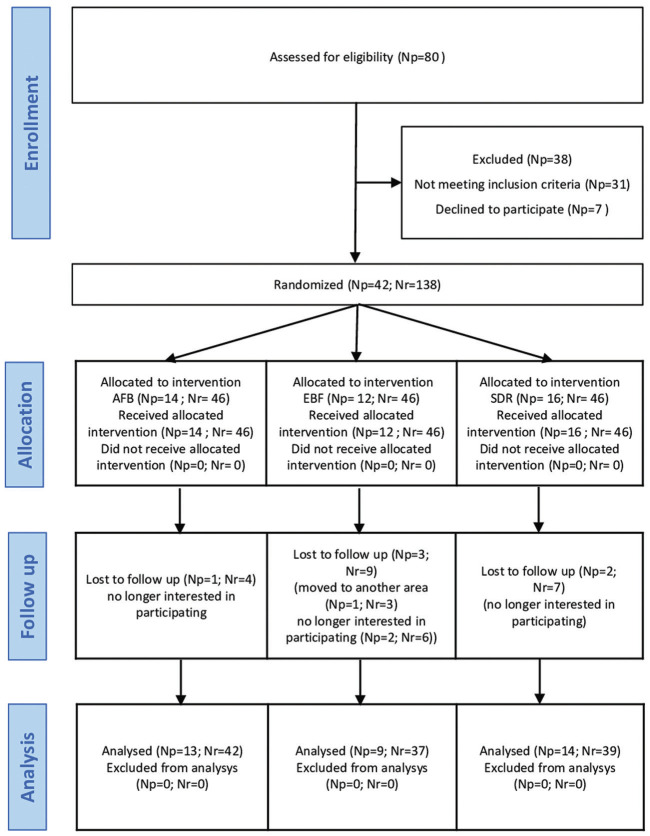
CONSORT flow diagram of the study (Np = number of patients; Nr = number of restorations).

With no exclusion of sex or race, 80 patients were assessed for eligibility. The enrollment of the participants was performed by an independent operator. Inclusion and exclusion criteria are listed in Table 2. All patients signed and returned a written informed consent about the clinical study, including the research procedure, materials, purpose, as well as risks and benefits and declared themselves willing to return at regular intervals for the complete evaluation period. After examination 38 individuals (47.5%) were excluded, either because they refused to attend the follow-up visits or because they did not meet the inclusion criteria. As a result, the study population was comprised 42 patients, 27 females (64.3%) and 15 (35.7%) males, aged from 18 to 61 years (with a mean age of 38 years). One hundred thirty-eight tooth preparations, divided up into 3 groups of n=46, were randomly restored by 2 experienced operators (A.S. and L.S.) blinded after assignment to interventions, as shown in Figure 1. The randomization was performed by putting letters in a sealed and opaque envelope, pointing out which restorative material would be used on each of the selected preparations. The envelope was opened by an independent operator, just before the restorative procedure began. The same operator assigned the participants to interventions. Each patient received at least three restorations, including one from each restorative material. If a volunteer had more than three teeth the selection of the cavities was randomized with the help of flipping a coin to select the material. Moreover, if the same tooth had 2 class III cavities that did not communicate at the mesial and distal sides of the tooth, both were included, respecting the upper limit of n=46 restorations per each material. All restorations were performed in the same clinical session. Details about the characteristics of the lesions included in the study and the distribution of the materials are presented in Table 3.

**Table 2:**
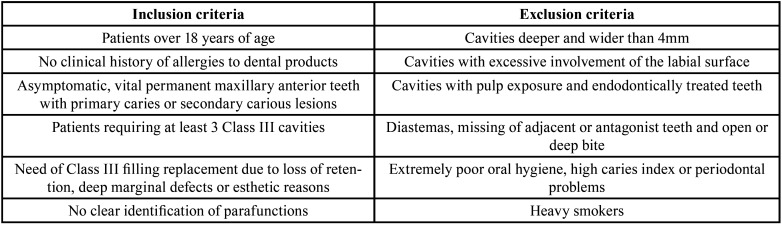
List of inclusion and exclusion criteria.

**Table 3:**
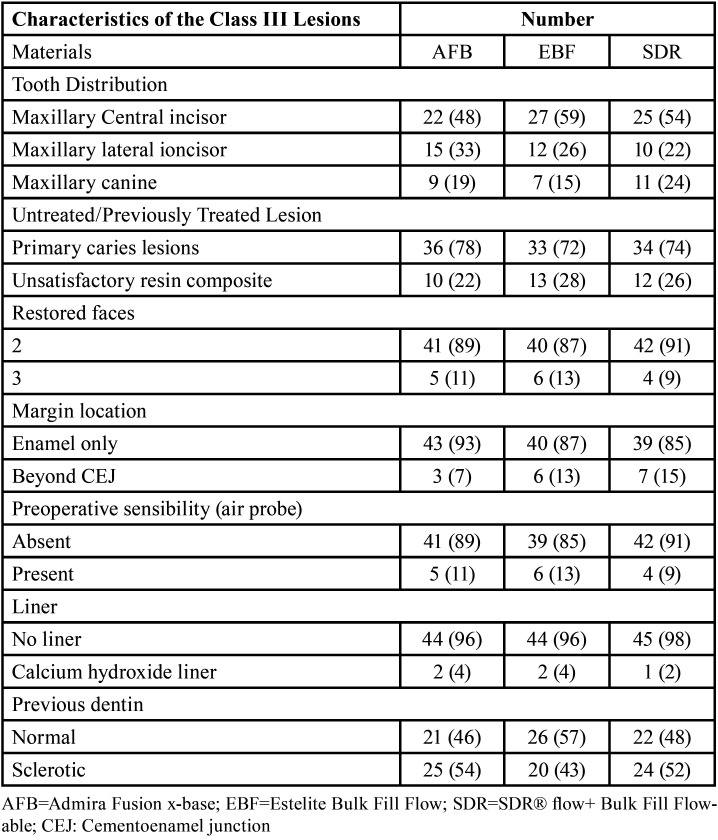
Baseline data regarding the lesions included in the study (% of tooth type).

-Restorative procedures:

The week preceding the restorative procedures the patients were submitted to supragingival dental scaling for a single session and received verbal oral hygiene instructions. At the treatment session initial periapical radiographs of the teeth to be restored were taken and before treatment, tooth sensitivity and the pulp health status were assessed by means of a thermal sensitivity test and the scores were then recorded. Later, in order to prevent discomfort during the restorative procedures, all patients received local anesthesia with articaine 1:100.000. The teeth to be restored received dental prophylaxis with a rubber cup, non-fluoridated pumice, and water at low speed, then dried with oil-free air, before shade selection, which was performed using a shade guide (VITA classical A1-D4, VITA Zahnfabrik, Bad Säckingen, Germany).

All the clinical procedures were performed by both operators with the aid of 4.3x400 surgical head-worn loupes (KS, Carl Zeiss Vision, Jena, Germany) and isolation of the working field by means of a rubber dam (Hygenic Latex Dental Dam, Whaledent Inc., Cuyahoga Falls, OH, USA) was performed for all restorations. To aim an adequate inversion of the rubber dam around the cervical area and in the gingival sulcus, knots ligatures with dental floss were performed.

Cavity preparations and removal of old unsatisfactory restorations were performed using round diamond burs (Komet/Brasseler, Lemgo, Germany) at high speed with water cooling and slow-speed tungsten round carbide burs and/or excavators were used to remove carious dentin. A conservative cavity design was used and no additional retentive features were prepared. Conversely, the preparation dimensions of the replaced restorations were inevitably imposed by the existing composite restoration and if present by the secondary carious lesion. For all restorations the preparation depth (from the buccal to the lingual aspect) and width (from the incisal to the cervical aspect), measured by means of a periodontal probe, was between 2 and 4 mm. However, the cavity dimension in each patient had mainly not the same size. In the case of a labial involvement of the preparation, a very short bevel (~=0.5mm) was performed to the labial margins only in order to avoid sacrificing enamel. The labial extension of the cavity was never more than 1 mm, in order to preserve labial enamel and to minimize the appearance of the restoration at the buccal side. The restorations involving the labial aspect of the tooth were limited, 5 (11%), 6 (13%), 4 (9%) for AFB, EBF, SDR respectively. Finally, the preparation was finished with a fine-grained diamond bur on a low-speed handpiece. The margins of the restorations were located in enamel, with the exception of 16 (22%) preparations in which the gingival margin was located beyond the cementoenamel junction (CEJ). Calcium hydroxide (Life, Kerr Italy, Scafati, Italy) as a cavity liner material over the deepest portion of the preparations with limited remaining dentine thickness was needed for 5 cavities (7%).

All preparations received the 2-step self-etch adhesive system Clearfil SE Bond (C-SE) (Kuraray, Osaka, Japan). However, a selective etching strategy was applied for all restorations. Thus, the enamel margins were etched with 37% phosphoric acid (DMG Chemisch-Pharmazeutische Fabrik GmbH, Hamburg, Germany) for 15 seconds, then thoroughly rinsed with an air/water spray for 15 seconds and gently air dried, leaving the dentin slightly moist. Afterwards the primer and the bonding were applied to the preparations according to the manufacturer’s instructions with an active brushing procedure. Then light cured for 10 seconds in the standard application mode at 1000 mW/mm2 by means of a calibrated light-emitting diode (LED) curing unit (Bluephase 20i, Ivoclar Vivadent, Schaan, Liechtenstein). Calibration of the LED light device was performed with a radiometer (Demetron; Kerr, Orange, CA, USA).

In order to restore the shape of the proximal walls, sectional metal matrices (Palodent, Dentsply Detrey GmbH, Konstanz, Germany) were placed and firmly fixed with wooden wedges. Burnishing of the matrix was done in order to improve the contact point. According to randomization, the three flowable bulk-fill resin composites were placed in the preparation following the manufacturer’s instructions, in a single bulk increment from bottom to top, keeping the tip immersed during application and finally adapted with dental composite brushes in order to achieve adequate shape and contour (Brush nr 24, Tokuyama, Tokyo, Japan). Then light cured for 40 seconds. In the event that air bubbles were detected, these were pierced with a clean explorer and excess material was removed prior to curing.

After removal of the rubber dam, the occlusion was evaluated with 40 µm articulating paper (Bausch, Köln, Germany) and excursive interferences in lateral, latero-protrusive, and protrusive excursions were adjusted with fine diamond burs. Final contouring, finishing and polishing procedures were performed at the same appointment with polishing silicon points (Identoflex, KerrHawe, Bioggio, Switzerland) and using consecutive polishing disks and finishing strips (Sof-Lex, 3M Oral Care, St. Paul, MN, USA) in order to shape of the proximal surface. Finally, polishing pastes (Shiny, Micerium, Avegno, Italy) were used with a low-speed dry brush. Post operatory periapical radiographs of the restored teeth were taken prior to dismissing the patient.

-Clinical evaluation:

Two experienced dentists other than the operators involved in the placement of the restorations were calibrated prior to the study and performed an independent assessment of the restorations during the follow-up. The 2 examiners were blinded to the restorative material used for any Class III cavity at all assessments. Calibration was carried out in two sessions on 20 class III restorations from 10 patients who were not participating in the present study. The intra-examiner agreement was 100% for all the evaluated criteria. When disagreement occurred during the evaluations, the restorations were re-evaluated by both examiners and a consensus was obtained before the patient was dismissed.

The restorations were evaluated at baseline (7 days) and at 6, 12, 18 and 24 months of clinical use according to slightly modified US Public Health Service (USPHS) Guidelines (24). The criteria variables are listed in Table 4. The overall clinical success rate was considered for the criteria of retention, marginal adaptation, marginal discoloration, and secondary caries. Partial retention and loss of retention, as well as restorations that needed to be repaired or replaced due to severe marginal defects or deep marginal staining and also the occurrence of secondary caries were considered to be clinical failures. At each recall, the evaluation was performed under isolation of the working field with cotton rolls and visual examination was carried out using 4.3x400 surgical head-worn magnification loupes. The postoperative sensitivity was evaluated by applying air for 10 seconds from a dental syringe at a distance of 1 cm from the tooth surface and by questioning the patients. During this evaluation, adjacent teeth were isolated with cotton rolls. Periapical radiographs were taken at the last 24-month recall.

**Table 4:**
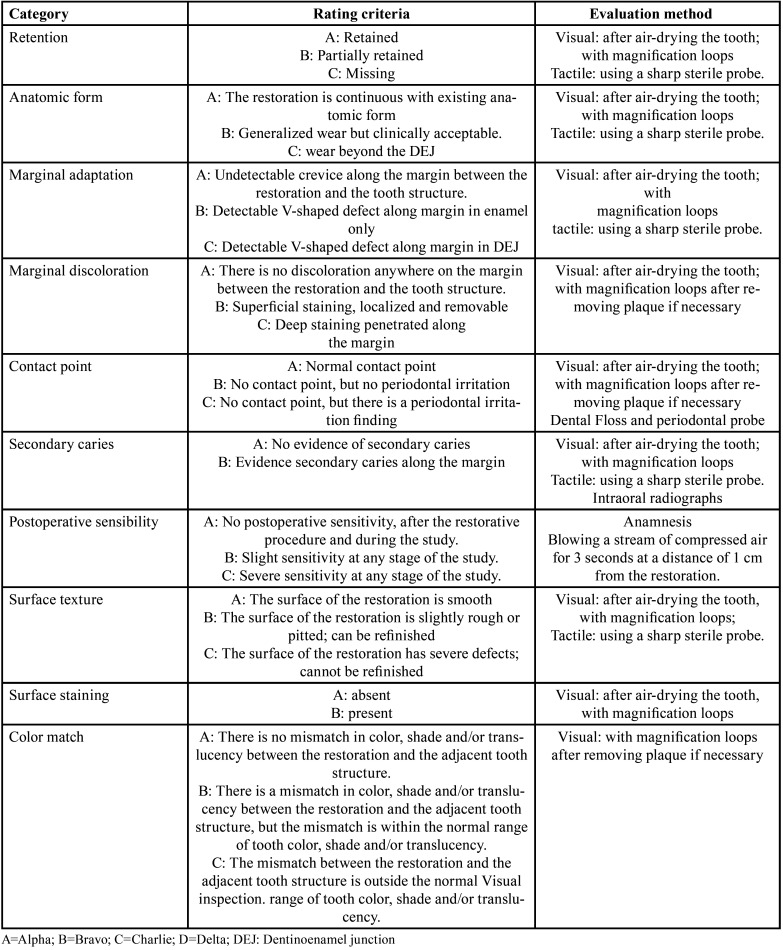
Modified USPHS clinical criteria rating system.

-Statistical analysis:

Cohen’s Kappa statistic was used to assess the degree of agreement between the two evaluators.

Concerning the answer to the first null hypothesis, the McNemar test was adopted to compare the success rate of each material between the baseline and at 24 months in terms of the considered clinical criteria. For the second null hypothesis, we used the Wilcoxon signed-rank test for two paired groups to compare, for each material, the ratings of the clinical criteria between the baseline and 24 months. Regarding the third null hypothesis, the Friedman test was used to compare the performance of the three materials at each time point in terms of the ratings of the selected clinical variables. For the binary criteria (secondary caries and surface staining), the differences among the materials were assessed with Cochran’s Q test.

All the tests were two-sided and a *p-value* lower than α = 0.05 was considered as evidence of statistical significance.

The data analysis was carried out with the R statistical software https://www.R-project.org/ (The R Foundation).

## Results

A total of 138 restorations placed in 42 patients were considered in the study. A limited number of patients were lost during the study due to reasons unrelated with the quality of the restorations. Drop-out rates of the restorations at 6, 12, 18 and 24 months were, respectively, 0%, 2.4%, 4.8% and 14.3%. A detailed description of the number of restorations lost during the follow-up for each material is displayed in the flowchart reported in Figure 1.

Cohen’s Kappa coefficients, computed for each clinical criteria, time point and material, ranged from 0.87 to 0.95, indicating a strong initial agreement between the two evaluators. The results for their clinical evaluations are presented in Table 5.

**Table 5:**
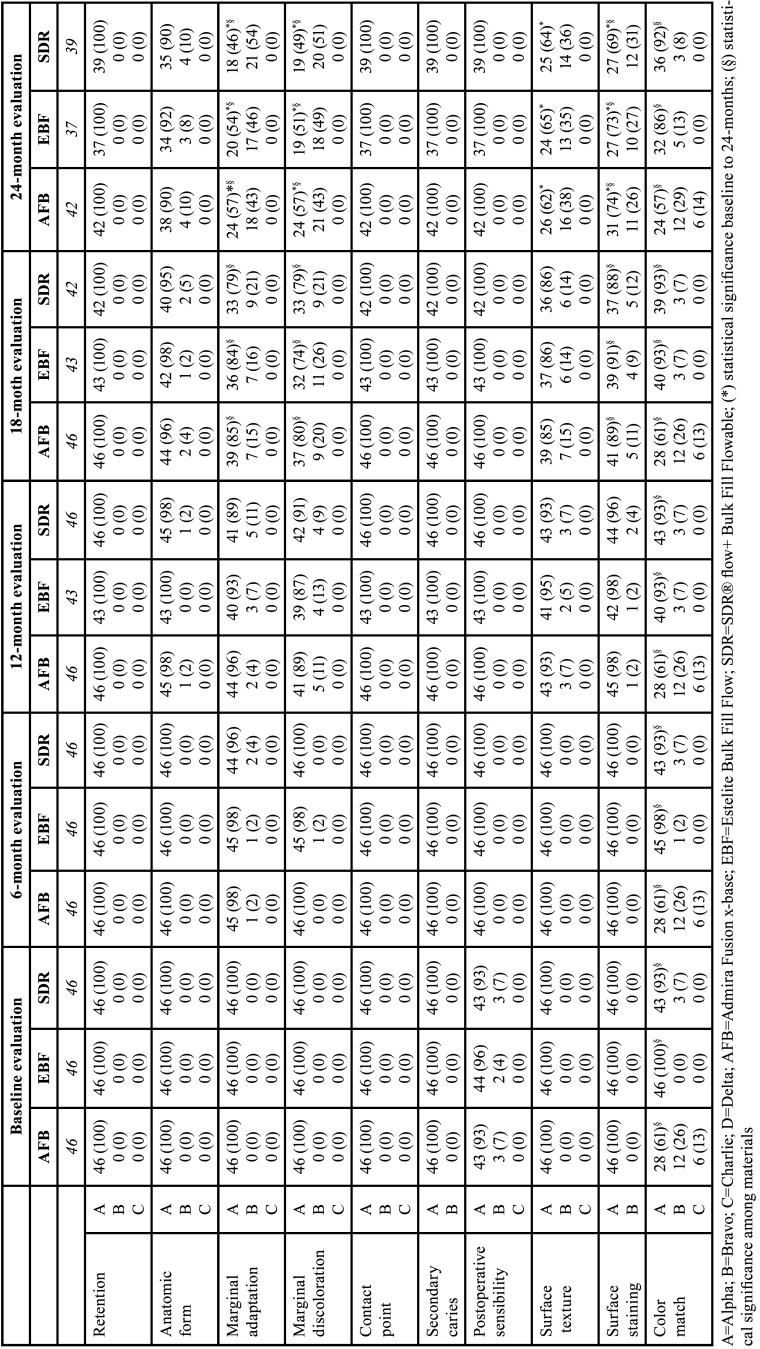
Rating frequencies (%) of the modified USPHS clinical criteria for the clinical evaluation of the restorations at baseline, 6, 12, 18 and 24 months.

The retention rate of the Class III restorations was 100% for each evaluated material since no restoration was missing or partially retained during the entire 24 months follow-up. Ratings of contact point, secondary caries and postoperative sensibility remained unchanged at the maximum (alfa) value during the entire follow-up for all the materials. Concerning the anatomic form, starting from the 12-month recall few restorations downgraded to the Bravo score, but without leading to significant differences according to the considered tests neither over time nor among materials. For all the selected clinical variables, the success rate at the 24-month follow-up was 100%. However, for the marginal adaptation, discoloration, surface texture and staining the Wilcoxon test returned a significant result due to the increased number of restorations rated Bravo at the end of the study for all the materials. Additionally, statistically significant differences among materials in terms of marginal adaptation, discoloration, and surface staining were detected with the Friedman test at 18 and 24 months. Finally, color match came out as the main discriminating factor among the materials. In fact, the Friedman test suggested that significant differences of the color match are present among the materials at each time point.

## Discussion

In the current clinical trial, the 24-month clinical performance of 3 flowable bulk-fill composites used in Class III cavity was evaluated. The recall rate was acceptable, as the chosen recall was consistent with the duration of the trial and the scheduled visits. The initiatives aimed at encouraging patient engagement were successful.

To assess in an objective and reliable way the clinical performance and to allow a comparison with similar clinical trials, a slightly modified USPHS clinical criteria were used (25). Similar previous clinical follow-up studies regarding restorations of Class III cavities adopted modified USPHS clinical criteria. However, the discriminative power of World Dental Federation (FDI) criteria has been reported as higher than the USPHS recommendations (26-28).

According to our findings, all Class III restorations were rated as clinically accepTable using the criteria of retention, marginal adaptation, marginal discoloration, and secondary caries and no restoration needed to be replaced, thus the 24-month overall clinical success rate was 100% for each tested material. Therefore, we failed to reject the null hypothesis.

To the best of the authors’ knowledge, this is the first study to evaluate the clinical performance of flowable bulk-fill composites used in Class III restorations. For this reason, it is not possible to make a direct comparison with earlier clinical trials, which used different restorative materials in Class III restorations such as macro-, micro- and nano-filled composites, hybrid composites, both regular viscosity or flowable, as well as compomers, glass ionomers and resin-modified glass ionomers (6,26,27).

In agreement with our findings, a 100% clinical success rate was previously reported in Class III restorations at 2 years (29), at 3 years (27) and even at 5 years (30-31). Moreover, a systematic review, that investigated the long-term survival of Class III restorations reported low annual failure rates, between 0% and 4.1% (3).

Likewise, a meta-analysis showed a 95% estimated overall success rate after 10 years for class III resin composite restorations (6). However the same authors advocated that the composite technology has limited or no impact on Class III restorations across different evaluation periods. In the light of this, Class III restorations are considered as the most durable resin composite restorations when compared to others (6).

Such high clinical success rates described in literature corroborate our results and might be attributed to several factors. The cavity is mainly surrounded by enamel, providing good marginal seal and mechanical retention (6,33). However, one of the major factors to be considered is the functional involvement of Class III restorations, basically placed in low-stress dental areas.

Advantage observed in the present study, which may contribute in reducing the chances of early failure, was the handling characteristics of the flowable bulk-fill materials tested. In fact, the clinical procedures for applying these materials in cavities are less technique sensitive and free of the conventional problems related to the use of contemporary micro-, nanofilled or hybrid resin composites, such as stickiness or multi-step application (15). The high success rate may also be attributed to the operator factor, considering that all restorative procedures have been performed by experienced practitioners.

The retention rates represent the most important evaluation criteria to determine the clinical success of restorative materials. In the current study, no restorations were lost during this 24-month clinical trial, resulting in a retention rate of 100% for all groups. As mentioned the low functional involvement, but also the cavity design and dimension may explain this clinical performance (21). Moreover, a low elastic modulus and an adequate curing at a 4mm thickness have been reported for these materials, which may have maximized the retention (18). Flowable bulk fill resin composites can act as elastic stress-absorbing material, since they have sufficient flexibility to resist polymerization shrinkage stress and favorably dissipate stresses such as thermal variations, water absorption and occlusal loads across the interface (17,20).

In previous studies, 100% retention rates were reported in Class III restorations, using the same bonding system applied in our clinical trial (7,34). The result was attributed to some extent to the bonding strategy. Differently from these findings, some authors do not identify a significant difference in retention rate in Class III cavities due to a different adhesive strategy, self-etch or total-etch (27,35).

Evidence shows efficacy of SE adhesives in the provision of optimal bond strength and high hydrolytic stability of the bonding on the dentine substrate (36-39).

Differently to dentin bonding, the adhesive performance at enamel of “mild” SE is still questioned. “Mild” SE adhesives are unable to etch enamel to the same depth as phosphoric acid due to their lower pH. Thus, enamel roughening and most importantly, selective enamel etching with phosphoric acid, is highly recommended prior their application (40-44). Accordingly C-SE was applied after an additional selective etching to enamel with 37% phosphoric acid, combining a more favorable etch-and-rinse treatment at enamel with a mild SE approach that appears to provide better long-term perspectives at dentin (45).

In the present clinical evaluation C-SE bond has been selected, due to the verified evidence of high performance in both in-vitro and in-vivo investigations (7,27,46,47).

Several studies evaluated the effect of additional etching of enamel on the clinical performance of Class III restorations bonded with C-SE (6,27). They concluded, that the same adhesive system used in our study, showed accepTable clinical performance and selective etching of the enamel cavity margins improved the marginal quality of restorations bonded with this adhesive system.

The aforementioned studies support of our findings, thus after a 24-month observational period no marginal failures in terms of marginal adaptation and marginal discoloration were detected. Actually, marginal quality scores of all tested restorative resin composites varied with respect to baseline measurements and significant differences among materials at the 18- and 24-month follow-up in terms of marginal adaptation and marginal discoloration was also detected. However, only minor evidence of a crevice along the margin into which the explorer could penetrate was visible and dentin was not exposed, making it a clinically acceptable situation. On the other hand, staining was superficial, located on an unspecific point on the enamel surrounding the restoration and easily removed by polishing procedures. With respect to these two clinical criteria, AFB presented the best clinical performance. In 16 (22,1%) cavity preparations the gingival margin was located below the cementoenamel junction (CEJ). However, the margin location did not negatively affect the clinical outcome.

Both clinical criteria, marginal adaptation, and marginal discoloration, depend on several factors, such as the effectiveness of the bonding system, or chemical/physical properties of the restorative material, as well as the operative technique, but these can be related to polymerization shrinkage also (6).

The low-viscosity bulk-fill resin composites containing lower filler volume demonstrated higher polymerization shrinkage values. However, an increase in the inorganic filler content can, to a certain extent, reduce the polymerization shrinkage due to the increased filler-to-monomer ratio (17,18).

In the present study differences of the filler content among the groups may only partially support this aspect.

The marginal adaptation *in vitro* studies of flowable bulk-fill materials has been reported as adequate (19,48-50). This effect can probably be explained by the low modulus of elasticity combined with the slower contraction rate of these materials, reducing the polymerization contraction stresses generated by the polymerization shrinkage and, thereby, maintaining the marginal integrity (11,51,52). In addition, flowability is regarded as a desirable handling property that allows for good wetting along the cavity walls, thus improving adaptation of the restorative material to the cavity walls and in fact, simplifying the operative procedure. This information may be a possible explanation for the positive results around all tested material margins after 24 months of the clinical evaluation. On the other hand, the flowability of these materials requires an accurate placement of the matrix, which has to be firmly fixed with wedges in order to re-establish the contour. The use of a clean explorer and of a dental composite brush also may help obtaining an adequate marginal adaptation.

In several clinical trials detectable margins and marginal staining, were observed more frequently than marginal caries, demonstrating that marginal degradation may lead to secondary caries lesions. However detecTable marginal gaps are not necessarily responsible for causing marginal caries and marginal discoloration is not indicative of marginal caries (5,53,54).

In the current study, no secondary caries lesions adjacent to the margins was detected in any of the restored teeth throughout the evaluation period (7). These findings are in accordance with previous studies, which reported, that secondary caries lesions adjacent to the restoration were infrequent in anterior dentition (3,6,30).

In this investigation, the color match with the surrounding dental tissue of the restoration was recoded as unacceptable in the AFB group only although at baseline evaluation EBF and SDR showed a good color match. Even if no significant difference from baseline to 24 month was observed, effectively a significant difference among materials was detected, as 13% of the AFB group scored Charlie at baseline. Thus, at the 24-month evaluation, the color stability for each tested material demonstrated an acceptable standard. The color mismatch at baseline of AFB may be attributed to the limited shade selection, as only a “universal” shade, corresponding to A2, was available. EBF and SDR offer to the clinician a wider shade option. Consistent with our findings, a color mismatch at baseline in Class III composite restorations was commonly observed (7,8,55).

It is worth noting, that in our study, the color match was not considered as a criterion for clinical success, since the restorations involving the labial aspect of the tooth was limited. An extended labial involvement of the cavity was considered an exclusion criterion. Consequently, a very short bevel of the labial enamel margins was prepared to preserve labial enamel and to avoid through and through cavities (56). In any case all patients presenting a restoration which scored Charlie were not disturbed by their appearance, as the restorations were not visible at the buccal side and the replacement was not considered to remedy the color mismatch.

In our study, all contact points remained unaltered, and slight deterioration in the surface texture and surface staining were observed among the evaluated groups. However, all restorations were considered clinically acceptable. Polishing procedures allowed to obtain smooth surfaces and to remove stains. The slightly rough surfaces may be caused by the wear, traceable to the restorative materials’ specifications, such as the filler type and content.

A minimal wear influenced the anatomic form as well, even if in a non-significant way. Typically, these materials have a lower filler content which renders the surface of the restoration less wear resistant. However, the filler content of all tested restorative resin composites, despite the differences, had a limited clinical impact after 24 months (15). Even if the wear resistance is a desired quality in posterior region, in Class III, also considering the limited extension of the restoration surface, it could be influenced by individual functional involvement of the teeth. On the other hand, surface staining can also be related to the patient’s habits and eating.

Regarding the postoperative sensitivity a slight non-spontaneous symptomatology after the restorative procedure, was reported only at baseline in 3 (7%) AFB, 1 (2%) EBF and 3 (7%) SDR restorations and totally disappeared at the 6 months recall. At 24 months there was no statistical difference between the experimental groups. This sensitivity found at baseline, caused by cold thermal stimuli only, was possibly associated with the restorative materials and procedures, but more probably with the trauma generated during the cavity preparation, regardless of the material used (57). All the seven preparations were deeper than the others and two of them received calcium hydroxide as a liner. Calcium hydroxide was only used in the deepest spots of the preparation for its bioinductive and antimicrobial activity. Our result is consistent with previous findings, that have exhibited progressive reduction in postoperative sensitivity using a mild 2-step self-etch bonding system most probably due to an adequate sealing of the dentinal tubules (27,58,59).

Despite the very high success rate observed for each of the tested materials, after a 24-month evaluation, statistically significant differences in terms of clinical effectiveness among each flowable bulk-fill were observed for several clinical criteria. Significant differences in Alpha and Bravo scores existed in marginal adaptation, marginal discoloration, surface texture, surface staining and color match, leading to rejection of the second and third null hypothesis.

As a concluding remark, the present clinical study has some limitations. All the clinical procedures were performed by experienced clinicians on a preselected sample. In addition, the material assignment was randomized with respect to the tooth type, but not with respect to the cavity location, namely mesial or distal. Moreover, the cavity dimension in each patient had mainly not the same size. The study limitations of this clinical trial may include the evaluation time, as a longer evaluation period could allow to recognize late failures and substantial differences in clinical performance between the tested materials. Our results may provide an indication for the future performance of flowable bulk-fill resin composites used in Class III cavities, but long-term clinical evaluations are required to fully explore their benefits and potential advantages in comparison with the conventional techniques. Although this clinical study covered a mid-term observational period of 24 months, the patients will continue to be followed-up with the aim of collecting long-term clinical data.

## Conclusions

During a 24-month follow-up period, the 3 tested flowable bulk-fill resin composites showed acceptable clinical performance, based on the criteria for the clinical evaluation of dental restorative materials. No clinical failure was observed, although significant differences were highlighted in marginal adaptation, marginal discoloration, surface texture, surface staining and color match. Within the limitations of this study, it can be concluded that the use of flowable bulk-fill resin composites as a restorative material in Class III cavities might be appropriate. However, further studies are required to confirm these encouraging results.
